# A common garden design reveals population‐specific variability in potential impacts of hybridization between populations of farmed and wild Atlantic salmon*, Salmo salar* L.

**DOI:** 10.1111/eva.12346

**Published:** 2016-01-27

**Authors:** Alison C. Harvey, Kevin A. Glover, Martin I. Taylor, Simon Creer, Gary R. Carvalho

**Affiliations:** ^1^ Molecular Ecology and Fisheries Genetics LaboratorySchool of Biological SciencesBangor UniversityBangorUK; ^2^ Institute of Marine ResearchBergenNorway; ^3^ School of Biological SciencesUniversity of East AngliaNorwichUK

**Keywords:** Atlantic salmon, farm escapees, growth, hybridization, temperature

## Abstract

Released individuals can have negative impacts on native populations through various mechanisms, including competition, disease transfer and introduction of maladapted gene complexes. Previous studies indicate that the level of farmed Atlantic salmon introgression in native populations is population specific. However, few studies have explored the potential role of population diversity or river characteristics, such as temperature, on the consequences of hybridization. We compared freshwater growth of multiple families derived from two farmed, five wild and two F1 hybrid salmon populations at three contrasting temperatures (7°C, 12°C and 16°C) in a common garden experiment. As expected, farmed salmon outgrew wild salmon at all temperatures, with hybrids displaying intermediate growth. However, differences in growth were population specific and some wild populations performed better than others relative to the hybrid and farmed populations at certain temperatures. Therefore, the competitive balance between farmed and wild salmon may depend both on the thermal profile of the river and on the genetic characteristics of the respective farmed and wild strains. While limited to F1 hybridization, this study shows the merits in adopting a more complex spatially resolved approach to risk management of local populations.

## Introduction

The long‐term evolutionary effects of both intentional and unintentional releases of domestic conspecifics on wild populations are of growing concern to conservationists and commercial forestry, fishing and wildlife stakeholders (Rhymer and Simberloff 1996; Laikre et al. 2010). Successful interbreeding between domestic and wild conspecifics may result in negative genetic effects such as loss of native population genetic structure, loss of genetic variation and the breakdown of local adaptations (McGinnity et al. 2003; Laikre et al. 2010). Fitness loss can occur when alleles important for local adaptation are replaced by maladaptive or nonlocal alleles through hybridization (observable in the F1 generation) (Randi 2008; Laikre et al. 2010) and by the loss of locally adapted gene complexes through introgression (generations of hybridization and backcrossing) (McClelland and Naish 2007), a mechanism of outbreeding depression. Ultimately, the local population genetic composition may be partly or completely replaced by that of the captive individuals (Sušnik et al. 2004; Meldgaard et al. 2007). Even if gene flow does not occur, native populations can be negatively affected by released individuals through direct competition for resources (Arechavala‐Lopez et al. 2011), potential disease transmission (Villanúa et al. 2008; Madhun et al. 2014) and wasted reproductive effort (i.e. nonviable offspring) (Rhymer and Simberloff 1996). Collectively, such impacts can lower native population productivity and may affect genetic diversity by decreasing effective population sizes (Hindar et al. 2006; Laikre et al. 2010). These detrimental ecological and genetic effects are particularly problematic for wild populations with low population sizes or at risk from extinction (Baskett et al. 2013).

A valuable tool for understanding the effects of interbreeding is to investigate how wild and farmed populations and their hybrids perform relative to each other when exposed to differing environments, for example using reaction norm studies (Leger and Rice 2003; Hutchings 2004; Darwish and Hutchings 2009). Reaction norms illustrate how a phenotype responds to environmental change, and such studies may expose genotype‐by‐environment (G × E) interactions, which can indicate that genetic variability for a phenotype or trait exists among conspecifics when exposed to different environments (Hutchings 2004). Common garden design studies, where individuals from all origins are reared communally and exposed to the same treatment(s) to eliminate random environmental effects, provide a way of investigating the genetic basis of phenotypic differences between groups (Hutchings 2011). Advances in molecular genetic technologies allow such comparative studies to elucidate genetic effects of hybridization and introgression for a variety of fitness‐related traits in disparate species (Fleming and Einum 1997; Leger and Rice 2003; Meldgaard et al. 2007; Colautti et al. 2009; Goedbloed et al. 2013). Within agriculture and forestry, studies on hybridization generally focus on crop–wild interactions and have provided valuable insights into how genetic changes within wild populations impact local plant population resilience and transgenic crop risk assessments (Adler et al. 1993; Viard et al. 2002; Mercer et al. 2007). In wildlife management, research has centred on interactions among wild and captive‐bred or feral conspecifics, with the aim of evaluating the risks of outbreeding depression in subsequent hybridized or introgressed populations (Walker et al. 2004; Randi 2008). From a fisheries perspective, the majority of hybrid–wild interaction studies have focussed on the effects of intentional stocking (Vasemagi et al. 2005; Hamasaki et al. 2010) or accidental escapes from commercial fish farms (McGinnity et al. 2003).

Arguably, the best studied species in terms of monitoring genetic impacts of escapees is the Atlantic salmon (*Salmo salar* L.). Recent decades have witnessed a marked increase in commercial production of Atlantic salmon in several countries with the global production of Atlantic salmon from aquaculture exceeding 2 million tonnes in 2012 (FAO 2014). Such rapid expansion has led to concern about potential negative environmental interactions imposed on native stocks by escaped farmed fish (Naylor et al. 2005; Weir and Grant 2005; Taranger et al. 2015). Ecological and genetic impacts of interactions between wild and farm escapees are compounded by difficulties in containing detrimental consequences due to the extent and scale of open marine systems (Naylor et al. 2000). Escape events are a common occurrence and often involve the accidental release of large numbers of farmed individuals (Soto et al. 2001; Morris et al. 2008; Norwegian Fish Directorate 2014, The Scottish Government 2014). In most countries where salmon farming is practised, it is a legal requirement to report any production losses; however, the reported numbers of escapees are most likely an underestimate of the true number as cases often go unreported (Glover et al. 2008, 2009a, 2010; Glover 2010; Taranger et al. 2015; Madhun et al. 2014). Catch statistics from experimental studies estimate that the number of escaped Atlantic salmon in the wild in Norway alone is in excess of a million individuals annually (Skilbrei et al. 2014).

The potential negative impacts of farmed fish on native populations stem from the genetic differences accrued in farmed stocks over the last few decades. Atlantic salmon aquaculture is based on rearing fish that originate from selective breeding programmes (Gjedrem et al. 1991; Gjedrem 2000, 2010). While a variety of commercially important traits have been selected for in domestic populations, growth rate and size have been the most consistently selected traits since breeding programmes were first initiated in the early 1970s (Gjedrem 2000). Growth in salmonids displays high heritability estimates, and the genetic gain for this trait has been estimated at 10 to 15% per generation (Gjedrem 2000). At present, the most advanced farmed populations have undergone more than 10 generations of directional selection, and as a result, their offspring display significantly higher growth rates than offspring of wild salmon under farmed conditions (Fleming and Einum 1997; Glover et al. 2009a; Solberg et al. 2013a,b). Furthermore, it has been observed that under farmed conditions, heritability estimates for growth are reduced in farmed relative to wild salmon (Solberg et al. 2012, 2013a). These results suggest the loss of genetic variation for growth, which is in accordance with genetic studies that have demonstrated reductions in allelic diversity at highly polymorphic genetic markers in farmed populations compared to wild conspecifics (Norris et al. 1999; Skaala et al. 2004; Solberg et al. 2012, 2013a). Body size is known to influence fitness and reproductive success in fish (Jonsson and Jonsson 2006; Garcia de Leaniz et al. 2007) and has been used as a proxy for fitness in other salmonid comparative studies (Einum and Fleming 1997; Solberg et al. 2013a). It is also known to influence the outcomes of resource and social competition (Post et al. 1999).

Wild Atlantic salmon are characterized by genetically distinct local populations, a product of their typically isolated freshwater habitats and their ability to home to their natal rivers to spawn (Taylor 1991; Carvalho 1993; Verspoor 1997; Garcia de Leaniz et al. 2007). The morphological and ecological divergence seen among wild salmon populations can to some degree reflect local adaptation to their native environments (Hindar et al. 1991; Taylor 1991; Carvalho 1993; Garcia de Leaniz et al. 2007; Houde et al. 2011; O'Toole et al. 2015) and likely underpin population resilience in changing environments (Hilborn et al. 2003; Schindler et al. 2010). Maintaining diversity both within and among populations can help to ensure the long‐term stability of populations against environmental change (Hilborn et al. 2003; Schindler et al. 2010). Several common garden studies have highlighted population‐specific genetic differences in early development in grayling (*Thymallus thymallus* L.) (Haugen and Vøllestad 2000) and brown trout (*Salmo trutta* L.) (Jensen et al. 2008), and between farmed and wild conspecifics and their hybrids or backcrosses for a variety of life‐history traits, including compensatory growth (Morris et al. 2011) and early development (Darwish and Hutchings 2009) in Atlantic salmon. However, there have been few studies that highlight the potential role of such population diversity on impacts of hybridization or introgression (Normandeau et al. 2009), and none under common garden conditions.

Recent studies have quantified introgression of farmed salmon escapees in 20 wild populations (Glover et al. 2012, 2013). These studies indicate that introgression levels are strongly population dependent and that the frequency of escapees is only modestly correlated with levels of introgression. Using a modelling approach on these empirical data, it has been subsequently demonstrated that population size, together with frequency of escapees, is a better predictor of introgression levels (Heino et al. 2015). Nevertheless, much of the variation in the levels of introgression of farmed salmon among native populations remains population specific: that is, the characteristics of each interacting farmed and native population may determine the degree and impacts of hybridization and introgression. While it has been suggested that the density of wild fish within an environment, and thus the level of competition between wild and farmed fish, is a significant factor influencing the relative success of farmed escapees among rivers (Glover et al. 2012, 2013), it is possible that other environmental or river‐specific factors may influence relative competitive success of farmed, hybrid and wild salmon in the wild. Water temperature is a key environmental factor that varies between rivers within and among regions. Temperature is also a key determinant of developmental and growth rates (Jonsson and Jonsson 2011; Forseth et al. 2011) and is therefore likely to be associated with adaptation of wild populations to natal rivers (Garcia de Leaniz et al. 2007). However, thus far, the relative variance in growth rate of different farmed salmon strains and wild salmon populations exposed simultaneously to a range of controlled temperatures has not been fully evaluated.

Studies that investigate genetic differences among farmed and wild conspecifics and their interaction in hybrid individuals are essential in understanding the mechanisms driving observed population‐level variance across a divergent set of environmental conditions. Understanding the potential effects of outbreeding depression and ecological interactions between farmed and wild conspecifics is necessary to underpin contemporary and future management strategies in a growing aquaculture industry, and for the formulation of conservation risk assessments (Randi 2008; Fraser et al. 2010; Laikre et al. 2010). Therefore, we investigated freshwater growth of multiple families derived from farmed, wild and hybrid salmon populations under three strongly contrasting temperature regimes to estimate variation in growth among populations. Three divergent temperatures were chosen to represent temperatures that approach the lower and upper boundaries for growth in Atlantic salmon and a temperature that is intermediate.

## Materials and methods

### Experimental crosses

**Figure 1:**
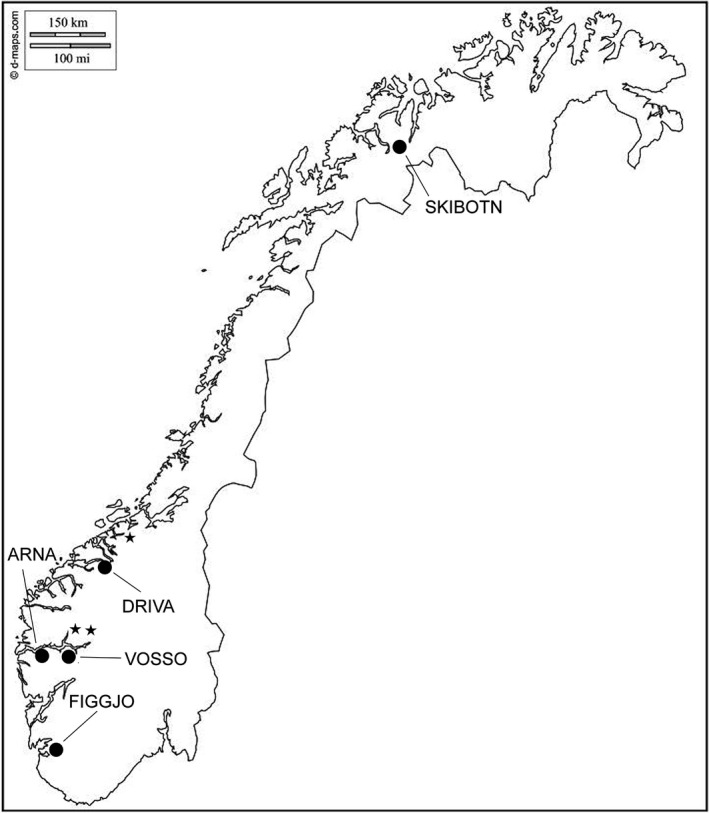
Map showing origin of wild populations. Wild fish collected from five river populations were included in this study. Gametes from the Vosso, Skibotn and Driva populations were collected from the Norwegian Gene Bank for Atlantic salmon. The * represents Haukvik, the Norwegain Gene Bank Hatchery, from which the Skibotn and Driva populations were collected, and the ** represents the Eidfjord Norwegian Gene Bank Hatchery where the Vosso population was collected.

**Figure 2:**
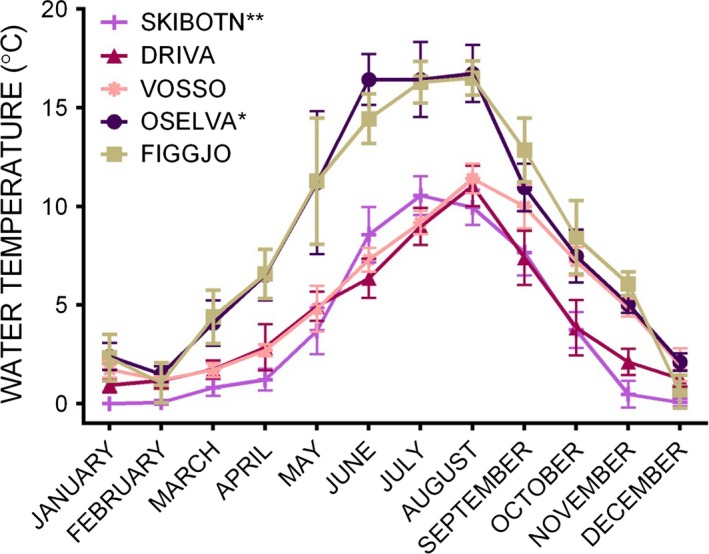
Average monthly water temperature for each of the rivers from which the experimental fish originated. Daily logger data from 2012 was used to calculate average monthly temperatures (and SD) within the rivers Figgjo, Oselva, Vosso and Driva. **Skibotn river water temperature was only available sporadically for years before 1986, and thus, the most complete data set (1986) was used to calculate average monthly water temperature in Skibotn. *There was no data available for the Arna River; thus, data from the nearby Oselva were used.

Adult brood fish collected from a total of five wild populations and two commercial farmed strains were used to produce the experimental families (Fig. 1). The two commercial farmed strains used were Mowi and Salmobreed. Mowi is the Marine Harvest strain and is the oldest Norwegian commercial strain (Gjedrem et al. 1991). Salmobreed was established in 1999 and is based on genetic material from several older Norwegian farmed strains. Both strains are extensively used in commercial aquaculture in Norway and internationally. Strain ID was not the focus here, and both were thus anonymized randomly as Farm 1 and Farm 2 and are referred to as the farm populations throughout. Wild parental fish upon which the families were produced were either sampled directly in rivers (Vosso, Figgjo, Arna) and verified as wild based on reading scale characteristics (Lund and Hansen 1991), or alternatively collected from the Norwegian Gene Bank for wild Atlantic salmon (Driva and Skibotn). The sire of family 17 had a tag when caught in the River Figgjo, which indicated that the specific fish originated from the nearby River Ims. The Norwegian Gene Bank is a programme that conserves wild salmon populations regarded as under threat from disease or extinction. Individuals are taken from the rivers and are then reared in the Gene Bank where genetic structure is monitored. Gametes from first‐ and third‐generation Driva and first‐ and second‐generation Skibotn gene bank strains were collected at the Gene Bank hatchery and transported back to Matre. Wild salmon from the River Figgjo (58°81′N, 5°55′E) are predominantly one‐sea‐winter fish with some two‐ and three‐winter fish (Friedland et al. 2009). The River Vosso (60°64′N, 5°95′E) is characterized by its large multi‐sea‐winter salmon, and the Norwegian Gene Bank conserves this population; thus, fish from this strain have been reared in a local hatchery before release into the fjord at the smolt stage. The River Arna (60°24′N, 5°29′E) is a small river in western Norway, with a variable‐age spawning population. The River Skibotn (69°38′N, 20°26′E) population in northern Norway is conserved by the Norwegian Gene Bank due to repeated infestation by the parasite *Gyrodactylus salaris*. The River Driva (62°40′N, 8°34′E) population in mid‐Norway is also conserved by the Norwegian Gene Bank due to infestation by *G. salaris*. Hydrographical data pertaining to river water temperature were accessed through the Norwegian Water Resources and Energy Directorate (2015). The average monthly water temperatures for each river are presented in Fig. 2. There was no data available for Arna; thus, the nearby Oselva River was used as a temperature reference. The highest temperature recorded was 16.7°C in Oselva, and the lowest recorded temperature was 0.0°C in Skibotn.

All 35 experimental families were established at the Matre experimental field station located on the west of Norway in weeks 46–47 of 2012. The five wild populations and two farmed populations were used to create farmed, F1 hybrid and wild families as follows: 8 farmed families consisting of Farm 1 and Farm 2, 8 hybrid families consisting of two F1 hybrid populations and 19 wild families consisting of fish from five wild populations. Figgjo females were crossed with Farm 1 males to produce the Hybrid 1 families, and Farm 2 females were crossed with Vosso males to produce the Hybrid 2 families. The full crossing design is presented in Table S1. All nine experimental groups are herein referred to as the experimental populations. All nine populations were represented by 4 families each with the exception of Driva, which consisted of just 3 families (Table S1).

### Experimental design

A common garden experimental design was used to investigate relative growth differences between farm, wild and hybrid F1 crosses of Atlantic salmon at three different temperatures. Salmon from a total of 35 families of farmed, wild and F1 hybrid origin were reared in communal tanks under standard hatchery conditions at three different water temperatures: the control treatment consisted of two replicate tanks at 12°C, while the treatments consisted of two replicate tanks at 7°C (low treatment) and 16°C (high treatment), respectively (Table 1). The temperatures were chosen to represent a representative range experienced by *S. salar* populations: 12°C is typically experienced by farmed salmon in a hatchery environment, 7°C and 16°C represent two contrasting temperatures experienced within the natural salmonid temperature range. Temperature regimes were maintained throughout the experiment, from transferral to tanks on 2 April until experiment termination on 23 to 27 September 2013. At the end of the experiment, individual growth measurements of wet weight and fork length were recorded and adipose fin or tail samples for DNA analysis were taken from a subset of individuals in each tank.

**Table 1:** Overview of the experimental design

Treatment	Low temperature (7°C)		Control temperature (13°C)		High temperature (16°C)	
Initial number of fish	Tank 1 (35 families of 30 fish = 1050)	Tank 2 (35 families of 30 fish = 1050)	Tank 3 (35 families of 30 fish = 1050)	Tank 4 (35 families of 30 fish = 1050)	Tank 5 (35 families of 30 fish = 1050)	Tank 6 (35 families of 30 fish = 1050)
Sampled	700	700	700	700	700	700
Genotyped	688	692	697	686	694	697

Two experimental replicates of each temperature treatment were performed, each initially containing 1050 fish; 30 fish from each of the 35 families; 700 fish from each replicate were sampled for genotyping. There were 12 outliers that were removed from the data set and 34 individuals that could not be unambiguously assigned to one family were also removed from the data set, leaving 4154 individuals in the analysis.

### Rearing conditions

Experimental replicates were established in week 4 of 2013, when 30 eyed‐eggs from each of the 35 families were sorted into six identical hatchery trays. (Thus, each replicate contained 1050 fish from the 35 families.) At the time of sorting, egg diameter was recorded for each family. In week 14, the hatched and ready‐to‐start feeding fry were transferred to six tanks, and the experiment was started. All tanks were 1 m in diameter and flow rate was 27 L/min. The fish were reared under standard hatchery conditions with a 24‐h light regime as per standard hatchery conditions. The fish were fed *ad libitum* with a commercial pelleted diet (Skretting), and pellet size was adjusted according to the manufacturer's tables, whereby a random sample of fish from each tank were measured at regular intervals to estimate average weight. Due to the high growth rate of fish at elevated temperatures, the high‐temperature replicates were split into two tanks each on 9 July; these were further split into 6 tanks on 8 August and then 8 tanks on 3 September. Thus, at the end of the experiment, each high‐temperature replicate consisted of 4 tanks. On 28 August, the control replicates were split into 2 tanks each. Mortality was low within all tanks, ranging between 8% and 12%.

### Sampling, genotyping and parentage assignment

Upon termination, 700 fish were randomly sampled from each of the replicate tanks (4200 in total). Where tank replicates had been split, an equal number of fish were randomly sampled from each split tank to make up a total of 700 fish per replicate. All individuals in each tank were euthanized following standard guidelines with an overdose of Finquel^®^ Vet anaesthetic (ScanVacc, Årnes, Norway). The fish were wet‐weighed, fork length measured and fin clipped for DNA analysis. Fins were placed individually into labelled tubes of 100% ethanol.

DNA‐based parentage testing was used to identify between 686 and 697 of sampled fish from each replicate back to respective family of origin. DNA was extracted in 96‐well plates using variation in the salt extraction method (adapted from Aljanabi and Martinez 1997). Parental DNA was extracted and genotyped twice to ensure consistent genotyping. Each plate contained 2 randomly placed negative controls (blank wells) to ensure unique identification of each plate. Five microsatellite loci were amplified in one PCR multiplex: *SsaF43* (Sanchez et al. 1996), *Ssa197* (O'Reilly et al. 1996), *SSsp3016* (GenBank # AY372820), *MHCI* (Grimholt et al. 2002) and *MHCII* (Stet et al. 2002). PCR products were resolved on an ABI Applied Biosystems 3731 Genetic Analyser and sized using a 500LIZ standard (Applied Biosystems). GeneMapper version 4.0 was used to score alleles manually. Individuals were then assigned back to family using the Family Assignment Program (FAP) (v3.6) (Taggart 2007), an exclusion‐based assignment program that is routinely used in other studies for the purpose of parentage assignment in comparative studies of salmonids (Solberg et al. 2013b; Glover et al. 2004; Skaala et al. 2013).

### Statistical analysis

Statistical analysis was carried out using R version 3.2.1(R Core Team 2015) with all critical *P*‐values set to 0.05. To test for differences in family representation among the split replicate tanks, a chi‐square (*χ* ^2^) test based on the numbers of fish in each family was performed. A linear mixed effect model (LME) was used to investigate the variation in weight at termination between the populations among the treatments, and covariates were analysed. The response variable was the continuous variable of log‐transformed weight at termination. Variation between split tanks and the replicate treatment tanks was controlled for by including split tank nested within replicates further nested within treatments in the model as random intercept factor effects with 14 levels. Variation within families across the treatments was controlled for by including family nested within strain as a random intercept effect (35 levels) with differing slopes for the effect of treatment.

#### Relative growth between the strains

The LME model was fitted using *lmer* from the *lme4* package in R (Bates et al. 2014). The full model was fitted with treatment (T) and population (P) as fixed factor covariates with 3 and 9 levels respectively, egg size (E) as a fixed continuous covariate, and all two‐way interactions between the fixed covariates: treatment and population (TP), treatment and egg (TE) and population and egg (PE) as fixed covariates, with tank (t) and family (f) as random covariates (as described above).

The fit of the full model was investigated by plotting the model residuals against all covariates, and the normality of the model residuals was visually confirmed using a histogram. The distribution of the random effects was investigated visually using quantile–quantile plots. The *lmerTest* package in R allows for automatic model selection using the *step* function (Kuznetsova et al. 2014). The function performs backwards selection on both the fixed and random effects to determine the simplest best fitting model (Kuznetsova et al. 2014).

It first performs backwards selection on the random elements of the model using likelihood ratio tests, with a significance level of 0.1 as a default, before performing backwards selection on the fixed elements in the model (Kuznetsova et al. 2014). The *anova* function from the *lmerTest* package was used to obtain *P*‐values for the fixed covariates of the model and are calculated using an *F*‐test based on Satterthwaite's approximation, and the significance level is set to 0.05 (Kuznetsova et al. 2014). The final model fit was confirmed by investigating plots of the model residuals against the covariates included in the model as well as those which were not included in the model. Normality of the final model residuals was confirmed visually using histograms. The full and final models, as given by the step function output, are presented in Table 2.

**Table 2:** Full model investigating weight variation (1.1)

Model	*N*	Response	Random effects				Fixed effects					
			Variable	Chi.sq	Chi Df	*P*	Variable	Sum Sq	Num Df	Den Df	*F*	*P*
1.1	4154	Log weight	**Tank**	69.41	1	**<1e−07**	**Treatment**	79.67	2	16.55	2292	**<1e−07**
			**Family**	157.04	5	**<1e−07**	**Population**	2.65	8	25	19	**<1e−07**
							**Egg**	0.09	1	25	4.9	**0.036**
							**T × P**	2.38	16	24	8.5	**0.000**
							**T × E**	0.17	2	25	4.9	**0.016**
							P × E	0.12	8	17	0.89	0.547

The variables in bold were retained in the final model, specified in (1.2). The interaction terms included in the full model: treatment: population (T × P), treatment: egg size (T × E) and population: egg size (P × E). *N*; number of individuals. Chi.sq, the value of the chi‐square statistics. Chi Df, the degrees of freedom for the test. *P*, the *P*‐values of fixed and random effects. Sum.Sq, sum of squares. Num Df, numerator degrees of freedom. Den Df, denominator degrees of freedom based on Sattherwaithe's approximations. *F*,* F*‐value.

Post hoc multiple comparisons were carried out for the interaction term of population by treatment using the function *pairs* in the *lsmeans* package with a Tukey's adjustment for multiple comparisons*,* which calculates the differences in least square means (Lenth 2015). The test computes all pairwise comparisons and reports *P*‐values and confidence intervals (Lenth 2015).

### Ethical statement

Temperatures experienced by the experimental fish were within the natural temperature ranges experienced by Atlantic salmon, and the rearing conditions where otherwise as in standard Atlantic salmon farming; therefore, approval of the experimental protocol by the Norwegian Animal Research Authority was not required. However, all welfare and use of experimental animals was performed in strict accordance with the Norwegian Animal Welfare Act 2010. In addition, all personnel involved in this experiment had undergone training approved by the Norwegian Food Safety Authority, which is mandatory for all personnel running experiments involving animals included in the Animal Welfare Act.

## Results

### Genotyping and parentage assignment

Of the 4200 individuals sampled, 34 individuals (<1% of the total) could not be assigned unambiguously back to a single family using the microsatellite multiplex and were removed from the data set prior to analysis. Twelve individuals were identified as outliers due to extreme condition factors attributed to human recording error and subsequently removed from the data set prior to analysis. The final data set for analysis contained a total of 4154 individuals.

### Statistical analysis

#### Growth between treatments

**Figure 3:**
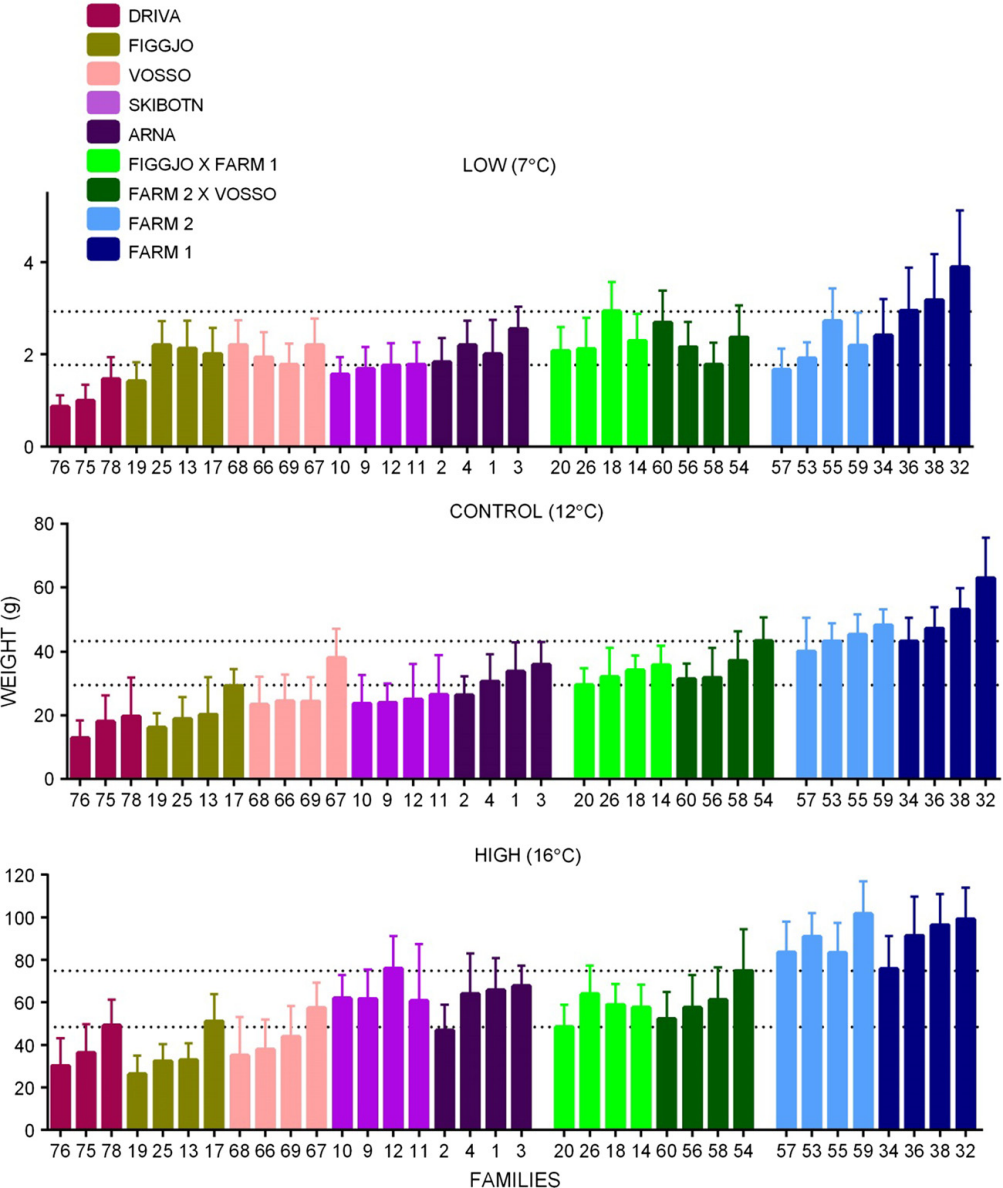
Average weight of each family within each population for the three treatment temperatures. The error bars represent the standard deviation. Certain families within populations performed better than other families within the same populations under certain temperature conditions. The populations performed differently across treatments. The dotted lines show the mean weight of the smallest and largest hybrid families. Hybrid crosses are labelled as maternal × paternal.

Final weight at termination was significantly different between each of the three treatments, being highest in the high‐temperature treatment, lowest in the low‐temperature treatment and intermediate in the control treatment (Table 2; Fig. 3). Growth in the low‐temperature treatment was very low for all strains, probably due to the low growth potential for salmon at this temperature. Within each split replicate, families were represented within their expected frequencies (*χ* ^2^ = 388.46, df = 442, *P* = 0.968), as expected with random sampling.

#### Relative growth differences between strains

The final model included all the covariates described above, apart from the interaction between population and egg size (Table 2). The fixed effect of population had a significant effect on weight at termination (Table 2). Adjusted pairwise comparisons between each population within each treatment are given in Table 3, with the significance level set to 0.05. On average, the farmed populations performed better than the hybrid and wild populations, while the hybrid performance was intermediate. It was evident, however, that some wild populations performed as well as or better than the hybrid and farm populations within particular treatments (Table 3; Fig. 3). The largest growth differences were seen between Farm 1 and Driva in the control temperature treatment where the farmed population grew three times more than the wild population. The smallest growth difference was observed in the low‐temperature treatment where the Farm 2 population growth was equal to both Arna and Vosso populations. In the control treatment, the smallest growth difference between farmed and wild populations was found between Farm 2 and Arna, where the relative growth ratio was just 1:1.4. The two farmed salmon populations were not significantly different from each other in growth rate in any treatment (Table 3). There was a visible, although not significant, trend in growth differences between the farm populations at the low temperature (Fig. 3). In the high‐temperature treatment, Skibotn and Arna had the highest wild population growth (Table 3). Driva grew significantly different to the other populations in at least one temperature treatment, apart from Skibotn (Table 3). On average, Driva displayed the lowest growth in the low‐ and control temperature treatments, while Figgjo had the lowest growth at high temperatures. The largest growth differences detected in the wild populations were between Arna and Driva where the relative growth ratio was 1:1.9 in the low and control treatments. Growth in the hybrid populations was not significantly different to each other in any treatment. Hybrid 1 displayed relatively intermediate growth to both its parental populations for all treatments (Table 3, Fig. 3). Hybrid 2 displayed similar relative growth to both its parental populations at low temperatures, while growth was intermediate between the parental populations in the other two treatments (Table 3, Fig. 3).

**Table 3:** (A) *P*‐values for the Tukey‐adjusted pairwise comparison of populations across treatments and (B) relative weight differences between each population at each treatment temperature

A	Driva			Figgjo			Skibotn			Vosso			Arna			Hybrid 1			Hybrid 2			Farm 2		
	7°C	12°C	16°C	7°C	12°C	16°C	7°C	12°C	16°C	7°C	12°C	16°C	7°C	12°C	16°C	7°C	12°C	16°C	7°C	12°C	16°C	7°C	12°C	16°C
Driva	–	–	–																					
Figgjo	**	ns	ns	–	–	–																		
Skibotn	ns	ns	ns	ns	ns	ns	–	–	–															
Vosso	**	.	ns	ns	ns	ns	ns	ns	ns	–	–	–												
Arna	**	***	ns	ns	ns	ns	ns	ns	ns	ns	ns	ns	–	–	–									
Hybrid 1	***	**	ns	ns	.	ns	ns	ns	ns	ns	ns	ns	ns	ns	ns	–	–	–						
Hybrid 2	***	**	ns	ns	.	.	ns	ns	ns	ns	ns	ns	ns	ns	ns	ns	ns	ns	–	–	–			
Farm 2	**	*	***	ns	**	*	ns	**	ns	ns	.	***	ns	ns	ns	ns	ns	ns	ns	ns	ns	–	–	–
Farm 1	*	*	***	ns	*	*	**	*	ns	ns	**	***	ns	.	ns	ns	ns	ns	ns	ns	ns	ns	ns	ns

B	Driva			Figgjo			Skibotn			Vosso			Arna			Hybrid 1			Hybrid 2			Farm 2		
	7°C	12°C	16°C	7°C	12°C	16°C	7°C	12°C	16°C	7°C	12°C	16°C	7°C	12°C	16°C	7°C	12°C	16°C	7°C	12°C	16°C	7°C	12°C	16°C
Ave. weight (g)	1.13	16.71	39.7	1.94	21.84	36.6	1.69	24.74	64.4	2.02	27.42	43.61	2.14	31.6	61.9	2.34	33.01	57.5	2.23	35.5	60.4	2.09	44.32	89.6
CV	0.40	0.55	0.38	0.31	0.42	0.37	0.27	0.40	0.28	0.27	0.37	0.39	0.29	0.27	0.26	0.29	0.20	0.21	0.30	0.25	0.29	0.33	0.18	0.17
Driva	–	–	–																					
Figgjo	1:0.8	1:0	1:1.2	–	–	–																		
Skibotn	1:1.1	1:0	1:0.9	1:0.9	1:0	1:1	–	–	–															
Vosso	1:0.7	1:0	1:1.3	1:0.9	1:0	1:1.4	1:0.7	1:0	1:1.4	–	–	–												
Arna	1:0.5	1:0	1:0.8	1:0.9	1:0	1:1	1:0.7	1:0	1:1	1:0.8	1:0	1:0.9	–	–	–									
Hybrid 1	1:0.4	1:0	1:0.7	1:0.9	1:0	1:0.8	1:0.7	1:0	1:0.8	1:0.8	1:0	1:0.7	1:1.1	1:0	1:0.7	–	–	–						
Hybrid 2	1:0.5	1:0	1:0.9	1:1	1:0	1:1.1	1:0.8	1:0	1:1.1	1:0.8	1:0	1:1	1:1.1	1:0	1:1	1:1.5	1:0	1:1	–	–	–			
Farm 2	1:0.2	1:0	1:0.5	1:1.1	1:0	1:0.6	1:0.8	1:0	1:0.6	1:0.9	1:0	1:0.6	1:1.2	1:0	1:0.6	1:1.7	1:0	1:0.6	1:1.3	1:0	1:1.1	–	–	–
Farm 1	1:0.2	1:0	1:0.6	1:0.1	1:0	1:0.7	1:0	1:0	1:0.7	1:1	1:0	1:0.7	1:1.3	1:0	1:0.7	1:1.8	1:0	1:0.7	1:0.9	1:0	1:0	1:0.1	1:0	1:0

The *P* values are represented as significance codes whereby ‘***’<0.0001, ‘**’<0.001, ‘*’<0.01, ‘.’≤0.5 and ‘ns’ denote not significantly different.

The treatments labelled as 7°C, 12°C and 16°C represent the low, control and high treatments, respectively. The populations were organized using the average weights from the control treatment and ordered from lowest average weight to highest average weight. Average weight of Farm 1 (not shown due to space constraints): 7°C: 3.13 g; 12°C: 51.49 g; 16°C: 90.8 g. Each population is compared with all other populations within each treatment. Ratios were calculated by dividing the average weights of the column populations by the row populations along the horizontal axis from right to left. Thus, the bigger fish were most commonly the numerator to ensure ratios of >1. CV: coefficients of variation for each population within each treatment. CV of Farm 1: 7°C: 0.36; 12°C: 0.22; 16°C: 0.20.

**Figure 4:**
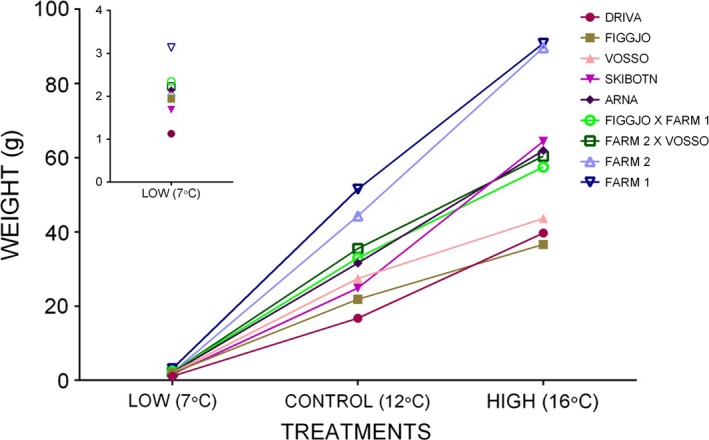
Growth reaction norms of each population. Average weight norms of reaction across the three treatment temperatures: low (7°C), control (12°C) and high (16°C). Replicate tanks have been pooled. The significant genotype‐by‐environment interaction is visible as the crossing lines between the populations across the treatments. For clarity, the inset graph represents the average weights of each population at the low‐temperature treatment.

To investigate further whether the observed differences in growth between the populations were changing between the treatments, an interaction term was included in the model. The interaction of population and treatment was retained in the final model (Table 2), indicating a population‐by‐temperature effect on final weight. Thus, the slopes of the reaction norms of the populations changed across the temperatures relative to the other populations, as evident in Fig. 4. The populations thus responded differently relative to each other to the different temperature treatments, indicating that population plays a role in salmon growth at varied temperatures.

A positive effect of egg size on final weight was detected; thus, families with a larger average egg size grew larger than those with a smaller average egg size (Table 2). The interaction between treatment and egg size had an effect on weight at termination (Table 2). Further analysis of the effect of egg size at the treatment level revealed that egg size was found to be a significant covariate in the low‐temperature treatment, and marginally insignificant in the high‐temperature treatment. The outputs, as given by the step function, for the models run to investigate significance of egg size on growth are presented in Table S2.

The above LME was run with population replaced by group (wild, farmed and hybrid) and all other covariates as presented above. The growth of the groups was significantly different between treatments. Farmed salmon were larger than both the hybrid and the wild salmon, with hybrid salmon displaying intermediate growth. In the low‐temperature treatment, farmed and hybrid growth did not differ significantly, although farmed salmon outgrew hybrids by 1.15 and both grew significantly more than wild salmon. The final model output, as presented by the step function, is given in Table S3.

## Discussion

In the present study, growth of two farmed, five wild and two F1 hybrid populations was investigated at three different temperatures using a common garden experimental design. Our study is the first to compare the growth of several different populations of wild, hybrid and farmed salmon in such an experimental setting across a temperature gradient. Overall, we found the following: (i) on average, farmed salmon outgrew wild salmon at all temperatures, with hybrids displaying intermediate growth; (ii) at the population level, there was significant variation among populations in growth rate to the extent that there was an overlap in weight between some wild populations and the hybrid and farm populations; (iii) there was a significant population‐by‐temperature interaction detected; and (iv) egg size (i.e. a maternal effect) was a significant predictor of size attained in the low‐temperature treatment but not in the control and high‐temperature environments.

### Temperature effects

For all populations, growth was greater in the high‐temperature treatment, intermediate in the control treatment and lowest in the low‐temperature treatment (Fig. 3). For most of the wild populations, the relative growth differences between the farmed and wild populations increased as the temperature increased (Table 3B), indicating that, although all the populations grew larger at higher temperatures, there are potentially larger growth differences between farmed and wild fish at higher temperatures, which may further influence the competitive balance between farmed and wild fish in rivers with warm thermal profiles, and may have implications for hybridization success under climate change.

To control for maternal effects, average family egg size was included in the LME. It was found that egg size was a significant predictor of growth at the low‐temperature treatment and marginally nonsignificant in the high‐temperature treatment. Such a pattern may derive from slow development at low temperatures whereby egg size influences early growth directly at this stage (Dunham 2004).

### Population effects

Populations investigated here are different to those used in previous growth studies; however, growth in some populations (Figgjo, Farm 1 and Hybrid 1) has been compared under different environmental parameters, and it displayed similar growth ratios to those seen previously (see Solberg et al. 2013b). Thus, the present study confirms earlier studies (Glover et al. 2009b; Solberg et al. 2013a), that growth in farmed salmon relative to wild salmon has been significantly increased through selection extending over ten generations in commercial breeding programmes. The magnitude of growth differences seen in our study is, however, on average, less than previously reported (Glover et al. 2009b; Solberg et al. 2013a,b). It is possible that the higher growth typical of farmed salmon under aquaculture conditions may further increase the growth differences observed between farmed and wild salmon due to competition interactions. Solberg et al. (2013b) investigated growth differences between farmed and wild conspecifics in mixed and single‐group tanks under controlled conditions. They found no difference in the relative growth across experimental designs, indicating that social interaction is not responsible for inflating the growth differences observed in aquaculture conditions (Solberg et al. 2013b).

### Population‐by‐temperature effects

On average, farmed salmon were significantly larger than wild salmon at all three experimental temperatures. However, when examined at the population level, the magnitudes of the growth differences were more variable than expected and influenced strongly by population (Table 3B, Fig. 3). Certain wild and hybrid strains grew well or better than other wild and farm populations in some of the treatments (Table 3B, Fig. 4). For example, while Farm 2 was larger than the wild and hybrid populations in the control and high treatments, certain wild and hybrid populations were larger, on average, than Farm 2 in the low‐temperature treatment (Fig. 4), and while Driva exhibited the lowest average growth overall in the low and control treatments, Driva outgrew Figgjo in the high‐temperature treatment (although this difference was nonsignificant after correction for multiple comparisons). Growth represents the genetic trait that has been documented to differ greatest between farmed and wild salmon, and previous comparative studies show that under aquaculture conditions, farmed salmon significantly outgrow wild salmon (Einum and Fleming 1997; Fleming and Einum 1997; Glover et al. 2009b; Solberg et al. 2013a,b). While the present study also found similar differences, significant growth variation was also detected among the wild populations (Table 3, Fig. 3). Hybrids displayed mostly intermediate growth relative to their respective parental populations (Table 3, Fig. 3). Intermediate hybrid growth relative to their parental populations has been documented for Atlantic salmon in comparative studies in aquaculture (Solberg et al. 2013a; Glover et al. 2009b), seminatural (Solberg et al. 2013b) and wild conditions (Einum and Fleming 1997). Intermediate manifestations of a variety of traits have been documented for other species, including Helmeted guineafowl (*Numida meleagris* L.) (Walker et al. 2004), sticklebacks (*Gasterosteus aculeatus*L.) (Hatfield and Schluter 1999) and eucalypts (*Eucalyptus* spp.) (Dungey et al. 2000).

Temperature plays an important role in maintaining adaptive population variation in developmental rates and survival in early life‐history stages in salmonid populations (Taylor 1991; Garcia de Leaniz et al. 2007). Studies have highlighted differences in populations for time of emergence and embryonic and larval survival that may be linked to local temperature regimes (reviewed in Taylor 1991). Temperature is also strongly linked to growth rates, which in turn influence important life‐history traits such as size and age at maturity and smolting (Jonsson and Jonsson 2006) and can influence competition (Post et al. 1999). Darwish and Hutchings (2009) investigated the genetic variation in early life‐history traits between farmed and wild backcrossed F2 Atlantic salmon under three different temperature regimes. They found genetic variation between populations for key life‐history traits such as time to hatch and posthatch survival. The results of the present study provide evidence for a genotype‐by‐environment interaction of an observable fitness‐related trait, namely growth across different temperatures. Thus, the competitive balance, exhibited as growth, between farmed and wild fish may be influenced by the origin of the farmed and wild fish.

Farmed salmon generally experience less variation in environmental parameters during production than wild salmon, such as low feeding competition, lack of predators and otherwise homogenous environmental conditions. During the early freshwater phase, for example during start feeding, the water temperature in the hatchery is typically elevated to 10 degrees or more in order to increase growth rates and produce a higher number of 0+ or 1+ smolts, depending upon the production strategy (Fjelldal et al. 2009). It could be expected therefore that farmed fish might not grow optimally in lower temperatures. Here, there was no evidence that the farmed fish grew any worse than expected at low temperatures; indeed, there was an overall lack of growth for all strains, and it is likely that the variability within and between the strains and the low growth observed derive from reduced growth across all strains. Thus, there was insufficient evidence found for thermal adaptation in the wild and farmed strains.

## Conclusions and recommendations

The population‐specific differences in growth demonstrated here represent analogous genetically based population diversity. Jensen et al. (2008) found population‐level differences in four wild brown trout populations for early life‐history traits at different temperatures and suggest that these populations are locally adapted to their native water temperature. While Jensen et al. (2008) focused on wild populations, Normandeau et al. (2009) compared gene expression of backcrossed Atlantic salmon farm–wild hybrids and their respective wild and farmed parent strains using 2 wild strains and 1 fourth‐generation farmed strain. They found significant population‐specific differences in liver gene expression of various transcripts between the strains and concluded that the consequences of introgression with farm genes will depend on the genetic architecture of the wild population (Normandeau et al. 2009). McGinnity et al. (2009) used a regression model to predict that influxes of hatchery genes into wild native salmon populations coupled with increasing water temperature due to climate change could negatively impact the local populations’ ability to adapt. Therefore, understanding how populations perform in variable temperatures is important for understanding how local populations might adapt to climate change (Jensen et al. 2008). Although limited to F1 hybridization effects, the present study clearly shows the merits in adopting a more complex spatially resolved approach to risk management of local populations. This is especially true of species where populations are likely to be locally adapted to their native environmental conditions or are at risk from outbreeding due to hybridization with nonlocal conspecifics. A study to investigate genetic structure in a historically genetically distinct lineage of grayling in the north Adriatic found that this critically threatened population has become heavily introgressed with a more homogenous Danubian grayling population due to indiscriminate stocking efforts (Sušnik et al. 2004). Gharrett et al. (1999) investigated outbreeding depression in F2 hybrids of pink salmon (*Onchorhynchus gorbuscha* W.) derived from two genetically distinct lines that are isolated based on even‐ and odd‐year life cycles. They found that fewer F2 hybrids survived relative to F2 controls (Gharrett et al. 1999). Although the studies above do not involve domestic vs. wild interactions, they serve to reinforce the potential negative effects of hybridization with genetically distinct or nonlocal populations.

Investigating the consequences of outbreeding and hybrid fitness on population integrity is vital to understand how wild populations will respond to hybridization and introgression over time (Fraser et al. 2010). While hybridization with novel farmed populations may initially cause an increase in genetic diversity (Glover et al. 2012), ultimately outbreeding depression via introgression may cause a loss of locally adapted wild population diversity and homogenization with farmed genotypes, which could threaten population stability and potential to adapt to ongoing environmental change. The success of introgression of escaped farmed salmon varies among rivers (Skaala et al. 2006; Glover et al. 2012, 2013). Thus, while our study focuses on F1 hybridization, the significant population‐by‐temperature interaction observed, coupled with natural variation in river temperature, may affect the level of hybridization and competition when there are large differences in body size between farm and wild conspecifics. Here, we found no differences in growth between the two hybrid populations for all temperatures, and their growth was intermediate between wild and farmed parental populations. Harbicht et al. (2014) investigated the effects of hybridization on adaptive potential after multiple generations of selection in the wild in transplanted combinations of wild, hybrid and domesticated brook trout (*Salvelinus fontinalis* M.) in new environments. Following several generations, it was concluded that introduced foreign genes were lost, and the hybrid populations came to resemble the wild population (Harbicht et al. 2014). Fraser et al. (2010) compared differences for a number of traits between farmed and wild Atlantic salmon and their multigenerational hybrids under common garden conditions. They found that wild backcrossing of hybrids did not completely restore trait distributions to their wild states (Fraser et al. 2010). Thus, the consequences of multiple generations of hybridization remain unclear, and further studies that investigate the effects of, specifically, multigenerational hybridization on population fitness are required.

Comparative studies that use Atlantic salmon as a model species to investigate the consequences of hybridization and introgression are important for the development of risk assessments and understanding of impacts for other aquaculture species. As aquaculture continues to expand worldwide through new production species, it will be important to focus on monitoring local populations to elucidate the integrity of genetic structure present and how escapees might affect this. Despite constraints arising from the limited number of families per population examined here and the incomplete range of farm–wild hybrid crosses, clear trends in performance were evident among populations across treatments. Thus, while we were able to document a G × E interaction, we acknowledge that further studies based on additional families and crosses would be beneficial. Studies that link the phenotypic differences observed in important life‐history traits to their underlying genetic structure, such as through linkage mapping, will likely advance management and conservation of both wild populations and their farmed conspecifics.

## Data archiving statement

We will not be archiving data as this manuscript does not have any associated data.

## Author contributions

GRC, KG and MIT conceived the study. AH performed all experiments and analysis and wrote the manuscript with input from GRC, MIT, SC and KG. All authors have read and approved the final manuscript.

## Supporting information


**Table S1.** Experimental crosses. Nine different populations were used to make three experimental groups: 8 farmed families consisting of two pure commercial populations; 8 hybrid families consisting of two F1 hybrid populations; and 19 wild families consisting of five wild populations. In this table and throughout the study the hybrid crosses are referred to as maternal × paternal.
**Table S2.** Full model investigating egg size variation between populations at the different treatment temperatures. The variables in bold were retained in the final models for each treatment. Egg size is only retained in the low‐temperature treatment. The interaction term represents population: egg size (P × E).
**Table S3.** Full model investigating weight variation where population is replaced by group. The variables in bold were retained in the final models for each treatment. The interactions included in the full model were: group: egg size (G × E), group: treatment (G × T), and treatment: egg size (T × E).
