# Supplementary material for: A common garden design reveals population‐specific variability in potential impacts of hybridization between populations of farmed and wild Atlantic salmon, Salmo salar L
Source: Evol Appl. 2016 Jan 27;9(3):435–49. doi: 10.1111/eva.12346 (PMC4778114; doi:10.1111/eva.12346)
Supplement: Supplementary file 1 — Table S1. Experimental crosses. Nine different populations were used to make three experimental groups: 8 farmed families consisting of two pure commercial populations; 8 hybrid families consisting of two F1 hybrid populations; and 19 wild families consisting of five wild populations. In this table and throughout the study the hybrid crosses are referred to as maternal × paternal. Table S2. Full model investigating egg size variation between populations at the different treatment temperatures. The variables in bold were retained in the final models for each treatment. Egg size is only retained in the low‐temperature treatment. The interaction term represents population: egg size (P × E). Table S3. Full model investigating weight variation where population is replaced by group. The variables in bold were retained in the final models for each treatment. The interactions included in the full model were: group: egg size (G × E), group: treatment (G × T), and treatment: egg size (T × E). [file EVA-9-435-s001.docx]

**Supplementary Material**

Table S1: Experimental crosses. Nine different populations were used to make three experimental groups: 8 farmed families consisting of two pure commercial populations; 8 hybrid families consisting of two F1 hybrid populations; and 19 wild families consisting of five wild populations. In this table and throughout the study the hybrid crosses are referred to as maternal x paternal.

| **Family** | Dam | Sire | Group | Population |
| --- | --- | --- | --- | --- |
| **1** | A1 | A9 | Wild | Arna |
| **2** | A2 | A10 | Wild | Arna |
| **3** | A3 | A11 | Wild | Arna |
| **4** | A4 | A12 | Wild | Arna |
| **9** | Ski1 | Ski3 | Wild/Genebank | Skibotn |
| **10** | Ski1 | Ski4 | Wild/Genebank | Skibotn |
| **11** | Ski2 | Ski3 | Wild/Genebank | Skibotn |
| **12** | Ski2 | Ski4 | Wild/Genebank | Skibotn |
| **13** | F1 | F11 | Wild | Figgjo |
| **14** | F1 | Farm1.11 | Hybrid | Figgjo x Farm 1 |
| **17** | F3 | F13 | Wild | Figgjo |
| **18** | F3 | Farm1.13 | Hybrid | Figgjo x Farm 1 |
| **19** | F4 | F14 | Wild | Figgjo |
| **20** | F4 | Farm1.14 | Hybrid | Figgjo x Farm 1 |
| **25** | F7 | F17 | Wild | Figgjo |
| **26** | F7 | Farm1.17 | Hybrid | Figgjo x Farm 1 |
| **32** | Farm1.1 | Farm1.11 | Farm | Farm 1 |
| **34** | Farm1.2 | Farm1.12 | Farm | Farm 1 |
| **36** | Farm1.3 | Farm1.13 | Farm | Farm 1 |
| **38** | Farm1.4 | Farm1.14 | Farm | Farm 1 |
| **53** | Farm2.3 | Farm2.11 | Farm | Farm 2 |
| **54** | Farm2.3 | V11 | Hybrid | Farm 2 x Vosso |
| **55** | Farm2.4 | Farm2.12 | Farm | Farm 2 |
| **56** | Farm2.4 | V12 | Hybrid | Farm 2 x Vosso |
| **57** | Farm2.5 | Farm2.13 | Farm | Farm 2 |
| **58** | Farm2.5 | V13 | Hybrid | Farm 2 x Vosso |
| **59** | Farm2.6 | Farm2.14 | Farm | Farm 2 |
| **60** | Farm2.6 | V14 | Hybrid | Farm 2 x Vosso |
| **66** | V2 | V10 | Wild/ranched genebank | Vosso |
| **67** | V3 | V11 | Wild/ranched genebank | Vosso |
| **68** | V4 | V12 | Wild/ranched genebank | Vosso |
| **69** | V5 | V13 | Wild/ranched genebank | Vosso |
| **75** | Dr2 | Dr7 | Wild/ranched genebank | Driva |
| **76** | Dr2 | Dr3 | Wild/ranched genebank | Driva |
| **78** | Dr5 | Dr7 | Wild/ranched genebank | Driva |

Table S2: Full model investigating egg size variation between populations at the different treatment temperatures. The variables in bold were retained in the final models for each treatment. Egg size is only retained in the low temperature treatment. The interaction term represents population: egg size (P x E).

|  | | | Random effects | | | | Fixed effects | | | | | | |  |
| --- | --- | --- | --- | --- | --- | --- | --- | --- | --- | --- | --- | --- | --- | --- |
| Model | N | Response | Variable | Chi.sq | Chi  Df | P | | Variable | Sum Sq | Num  Df | Den  Df | F | P | |
| Low (7°C) | 1380 | Log  Weight +1 | **1\|Tank** | 55.47 | 1 | **<0.000** | | P x E | 0.064 | 8 | 17 | 0.52 | 0.827 | |
|  |  |  | **1\|Fam** | 133.89 | 1 | **<0.000** | | **Population** | 1.306 | 8 | 25 | 11.26 | **0** | |
|  |  |  |  |  |  |  | | **Egg** | 0.117 | 1 | 25 | 7.6 | **0.010** | |
|  |  |  |  |  |  |  | |  |  |  |  |  |  | |
| Control (12°C) | 1383 | Log  Weight +1 | **1\|Tank** | 11.42 | 1 | **0.001** | | P x E | 0.062 | 8 | 17 | 0.36 | 0.928 | |
|  |  |  | **1\|Fam** | 105.07 | 1 | **<0.000** | | **Population** | 3.554 | 8 | 26 | 20.63 | **<0.000** | |
|  |  |  |  |  |  |  | | Egg | 0.018 | 1 | 25 | 0.84 | 0.368 | |
|  |  |  |  |  |  |  | |  |  |  |  |  |  | |
| High (16°C) |  | Log  Weight +1 | **1\|Tank** | 3.32 | 1 | **0.068** | | P x E | 0.129 | 8 | 17 | 0.96 | 0.497 | |
|  |  |  | **1\|Fam** | 135.27 | 1 | **<0.000** | | **Population** | 1.821 | 8 | 26 | 13.47 | **0.000** | |
|  |  |  |  |  |  |  | | Egg | 0.071 | 1 | 25 | 4.22 | 0.051 | |

Table S3: Full model investigating weight variation where population is replaced by group. The variables in bold were retained in the final models for each treatment. The interactions included in the full model were: group: egg size (G x E), group : treatment (G x T), and treatment : egg size (T x E).

|  | | | **Random effects** | | | | **Fixed effects** | | | | | |  |
| --- | --- | --- | --- | --- | --- | --- | --- | --- | --- | --- | --- | --- | --- |
| **Model** | N | Response | Variable | Chi.sq | Chi  Df | P | Variable | Sum Sq | Num  Df | Den  Df | F | P | |
| Group effects | 4154 | Log  Weight | **1\|Tank** | 69.38 | 1 | **<0.000** | G x E | 0.027 | 2 | 28 | 0.76 | 0.475 | |
|  |  |  | **1\|Family** | 432.35 | 1 | **<0.000** | **Group** | 0.87 | 2 | 31 | 25.24 | **0.00** | |
|  |  |  |  |  |  |  | **Treatment** | 53.03 | 2 | 26 | 1526 | **<0.000** | |
|  |  |  |  |  |  |  | Egg size | 0.057 | 1 | 31 | 3.26 | 0.081 | |
|  |  |  |  |  |  |  | **G x T** | 0.63 | 4 | 30 | 9.1 | **0.0001** | |
|  |  |  |  |  |  |  | **T x E** | 0.25 | 2 | 31 | 7.13 | **0.003** | |
